# Pulsating Extremely Low-Frequency Electromagnetic Fields Influence Differentiation of Mouse Neural Stem Cells towards Astrocyte-like Phenotypes: In Vitro Pilot Study

**DOI:** 10.3390/ijms25074038

**Published:** 2024-04-04

**Authors:** Jasmina Isaković, Filip Slatković, Denis Jagečić, Dražen Juraj Petrović, Dinko Mitrečić

**Affiliations:** 1 Omnion Research International d.o.o., 10000 Zagreb, Croatia; slatkofilip@gmail.com; 2 Laboratory for Stem Cells, Croatian Institute for Brain Research, University of Zagreb School of Medicine, 10000 Zagreb, Croatia; 3 Department of Histology and Embryology, University of Zagreb School of Medicine, 10000 Zagreb, Croatia; 4 Genos d.o.o., Laboratory for Glycobiology, 10000 Zagreb, Croatia

**Keywords:** extremely low-frequency electromagnetic fields, neural stem cells, differentiation, astrocytes, neurons, neuroregeneration

## Abstract

Even though electromagnetic fields have been reported to assist endogenous neurogenesis, little is known about the exact mechanisms of their action. In this pilot study, we investigated the effects of pulsating extremely low-frequency electromagnetic fields on neural stem cell differentiation towards specific phenotypes, such as neurons and astrocytes. Neural stem cells isolated from the telencephalic wall of B6(Cg)-Tyrc-2J/J mouse embryos (E14.5) were randomly divided into three experimental groups and three controls. Electromagnetic field application setup included a solenoid placed within an incubator. Each of the experimental groups was exposed to 50Hz ELF-EMFs of varied strengths for 1 h. The expression of each marker (NES, GFAP, β-3 tubulin) was then assessed by immunocytochemistry. The application of high-strength ELF-EMF significantly increased and low-strength ELF-EMF decreased the expression of GFAP. A similar pattern was observed for β-3 tubulin, with high-strength ELF-EMFs significantly increasing the immunoreactivity of β-3 tubulin and medium- and low-strength ELF-EMFs decreasing it. Changes in NES expression were observed for medium-strength ELF-EMFs, with a demonstrated significant upregulation. This suggests that, even though ELF-EMFs appear to inhibit or promote the differentiation of neural stem cells into neurons or astrocytes, this effect highly depends on the strength and frequency of the fields as well as the duration of their application. While numerous studies have demonstrated the capacity of EMFs to guide the differentiation of NSCs into neuron-like cells or β-3 tubulin+ neurons, this is the first study to suggest that ELF-EMFs may also steer NSC differentiation towards astrocyte-like phenotypes.

## 1. Introduction

For many neurological diseases, specific therapy still does not exist (e.g., stroke) or is unsatisfactory (e.g., multiple sclerosis, Parkinson’s disease). Thus, much of preclinical research and clinical trials is focused on testing and defining new therapeutic approaches. Among several of them, stem cells and externally applied electromagnetic fields have attracted a lot of attention. Stem cell research, including our work, has reported beneficial effects of transplanted stem cells on stroke, both in animal models [1] and clinical trials [2]. Since most cells disappear shortly after transplantation, it is reasonable to assume that their differentiation status plays an important role in determining their ongoing benefits.

Extremely low-frequency electromagnetic fields (ELF-EMFs) are vector fields that are generated during the displacement of electrically charged objects. Their main characteristics are that they exert a force on electric charges and interact with electromagnetic fields in their vicinity. They occupy the lower part of the electromagnetic spectrum, with their frequency ranging between 0 and 3000 Hz. Due to the innate charge of biological tissue, which makes it susceptible to electromagnetic field influence [3,4,5], research into the effects of EMFs on brain development and neuroregeneration has entered the spotlight. Previous studies have confirmed that ELF-EMFs can promote adult hippocampal neurogenesis through the upregulation of the proliferation and differentiation of NSCs into neurons, both in vitro and in vivo [6,7,8]. On the other hand, studies quantifying the impact of ELF-EMFs on the promotion of NSC differentiation towards the astrocytic phenotype are lacking. Since astrocytes are one of the crucial players within the neuro-immune axis, playing a role in sustaining tissue regeneration or further damage, it is vital to quantify the effects of EMFs on this cell type.

Given the significance of neuroectodermal cell differentiation and migration post-transplantation, and the reported beneficial impact of electromagnetic fields on nervous tissue conditions in numerous studies [9,10,11,12], this pilot study aims to investigate the effects of pulsed ELF-EMFs on the differentiation of NSCs into astrocytes and neurons. The obtained results demonstrate significant alterations in NSC differentiation pathways following EMF exposure, most notably towards astrocyte-like phenotypes. Considering the limited availability of neuronal precursors in the human subventricular zone and the hippocampus, the demonstrated impact of electromagnetic fields on stem cell characteristics in this study could potentially contribute to understanding the observed beneficial effects of EMF application on neural tissue, as reported in previous studies [9,10,11,12]. Therefore, this in vitro study significantly broadens the scope for advancing to in vivo studies wherein transplanted stem cells could be modulated by EMFs, thereby enhancing their therapeutic efficacy.

## 2. Results

To test the influence of extremely low-frequency electromagnetic fields on neural stem cell fate, we used the following markers: NES, a marker of undifferentiated progenitor state; β-3 tubulin, a marker of neuronal fate; and GFAP, a marker of astrocytic fate. We exposed three dedicated groups of cells to three different electromagnetic field strengths: $B_{1}=1.21–7.26\;mT$; $B_{2}=7.26–13.31\;mT$; $B_{3}=13.31–18.15\;mT$. Each of these was applied for 1 h, with a frequency of 50 Hz.

### 2.1. No Influence on Cell Number

**Figure 1:**
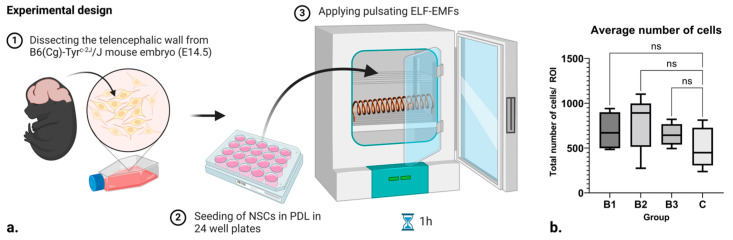
(**a**) Scheme of the experimental design and setup. (**b**) Box and whisker plot showing the number of cells within each respective control and treatment group, where B_1_ = 1.21–7.26 mT, B_2_ = 7.26–13.31 mT, and B_3_ = 13.31–18.15 mT. Here, ROI stands for “Region of Interest”, while ns denotes “non significance”. Created with BioRender.com (accessed on 7 February 2024).

Since certain frequencies and strengths of electromagnetic fields can induce cell death, the average number of cells in each group was tracked. No difference was observed (Figure 1b). This is a good indicator that the applied ELF-EMFs did not induce a significant number of cytotoxic effects.

### 2.2. Upregulation in NES Expression

**Figure 2:**
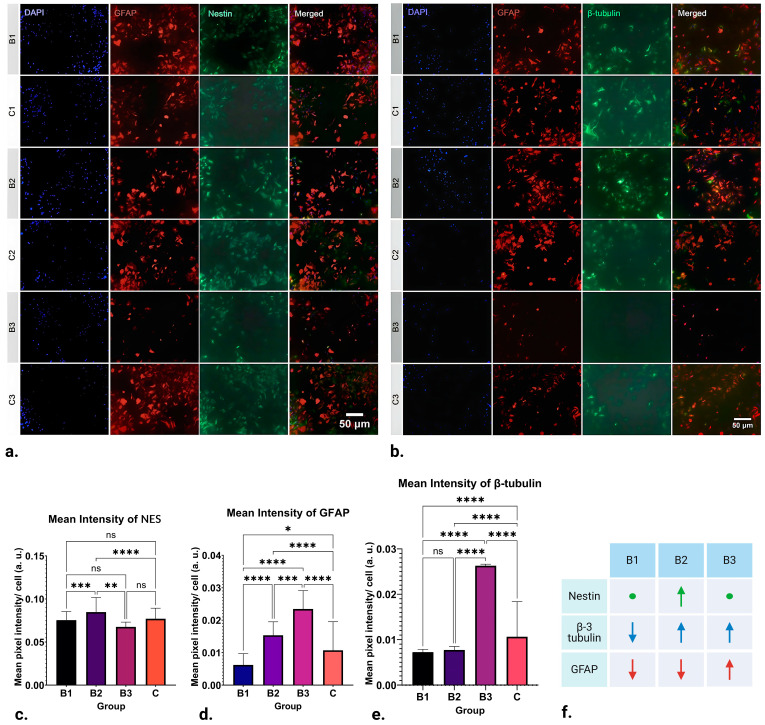
Results of ICC staining of differentiating NSCs treated with ELF-EMFs of strengths B1 (1 h; 1.21–7.26 mT; 50 Hz), B2 (1 h; 7.26–13.31 mT; 50 Hz) and B3 (1 h; 13.31–18.15 mT; 50 Hz), and control groups. (**a**) ICC staining for DAPI, GFAP, and NES. (**b**) Representative ICC staining for DAPI, GFAP, and β-3 tubulin. (**c**) There is no significant difference in NES expression between the control, B1, and B3 groups. Significant upregulation in the marker’s expression can be seen in group B2 (*p* ≤ 0.0001). (**d**) Downregulation of β-3 tubulin expression in B1 and B2 groups can be observed (*p* ≤ 0.0001). On the other hand, β-3 tubulin expression appears to be significantly upregulated in group B3 when compared to the control (*p* ≤ 0.0001). (**e**) GFAP expression appears to be downregulated in group B1 (*p* ≤ 0.05) and significantly upregulated in groups B2 and B3 when compared to the control (*p* ≤ 0.0001). (**f**) Schematic of the obtained results. As compared to the control, arrows pointing up represent upregulation, arrows pointing down represent downregulation and circles represent no change in NES (green), β-3-tubulin (blue), and GFAP (red) expression in ICC. * *p* ≤ 0.05; ** *p* ≤ 0.01; *** *p* ≤ 0.001; **** *p* ≤ 0.0001, ns denotes “non significance”. Created with BioRender.com (accessed on 7 February 2024).

The results of ICC analysis on NES expression in NSCs are visible in Figure 2a,c, where the mean value recorded in groups exposed to B1 (1 h; 1.21–7.26 mT; 50 Hz) and B3 (1 h; 13.31–18.15 mT; 50 Hz) demonstrate no significant difference in expression. On the other hand, significant upregulation in NES expression can be seen in groups exposed to B2 (1 h; 7.26–13.31 mT; 50 Hz) when compared to the control (*p* ≤ 0.0001).

### 2.3. Upregulation and Downregulation of GFAP Expression

The results of ICC analysis of GFAP expression in NSCs are depicted in Figure 2a,d. There was a distinct variation in the expression of GFAP where EMFs of strength B1 (1 h; 1.21–7.26 mT; 50 Hz) were applied, leading to its downregulation (*p* ≤ 0.05). Nevertheless, the most noteworthy observations are found in groups B2 (1 h; 7.26–13.31 mT; 50 Hz) and B3 (1 h; 13.31–18.15 mT; 50 Hz). In these groups, the application of EMFs significantly upregulated GFAP expression (*p* ≤ 0.0001). In other words, expression of GFAP appears to be inversely proportional to the strength of the applied fields: it is downregulated by weaker fields and upregulated by stronger ones.

### 2.4. Upregulation and Downregulation of β-3 Tubulin Expression

After statistical analysis of the results obtained for the intensity of labelling for the neuronal marker β-3 tubulin, striking differences can be found in all three runs, as seen in Figure 2b,e. Namely, the expression of β-3 tubulin in group B1 (1 h; 1.21–7.26 mT; 50 Hz) appears to be significantly downregulated by EMF application (*p* ≤ 0.0001). A similar pattern of downregulation is also observed in B2 (1 h; 7.26–13.31 mT; 50 Hz) (*p* ≤ 0.0001). Contrastingly, the application of EMFs in group B3 (1h; 13.31–18.15 mT; 50 Hz) appears to cause a significant upregulation in β-3 tubulin expression (*p* ≤ 0.0001). Observing this pattern, it can be concluded that β-3 tubulin expression appears to be upregulated by higher-strength ELF-EMFs while being downregulated by those of lower strength.

## 3. Discussion

Although EMFs have been shown to influence the regeneration of nervous tissue, little is known about how they influence astrocytes, and only some data on their influence on neurons exist. Thus, in this in vitro pilot study, we have studied the influence of pulsating extremely low-frequency electromagnetic fields on differentiating neural stem cells in vitro to determine how EMFs influence the expression of some major markers of neuronal fate. Indeed, our findings indicate that the expression of NES, GFAP, and β-3 tubulin is significantly influenced by the strength of the applied ELF-EMFs (Figure 2f).

### 3.1. Pulsating ELF-EMF Exposure and Potential for NSC Differentiation towards the Neuronal Phenotype

Interestingly, while EMFs of strength 1.21–7.26 mT (B1) caused a downregulation in β-3 tubulin expression (*p* ≤ 0.0001), those of 13.31–18.15 mT (B3) induced its significant upregulation (*p* ≤ 0.0001) (Figure 2e). Given that β-3 tubulin, a key component of microtubules, plays a pivotal role in maintaining axon guidance, it is suggested that pulsating 50 Hz ELF-EMFs could have a profound impact on in vitro neuronal differentiation, potentially facilitating or obstructing endogenous neurogenesis. This hypothesis underscores the intricate interplay between ELF-EMFs and neuronal development, highlighting the potential for both the enhancement and suppression of differentiation.

β-3 tubulin, also known as βIII-tubulin, is a microtubule element found almost exclusively in neurons and in testis cells. It is one of the seven β-tubulin isotypes identified in the human genome. Microtubules, composed of α- and β-tubulin heterodimers, play critical roles in cellular processes, including vesicular transport, cell motility, and mitosis [13]. The expression of β-3 tubulin is tightly restricted, and aberrant expression is associated with drug resistance and aggressive disease [13]. The differential expression of tubulin isotypes is particularly prominent during development and in specialized cells, suggesting that some isotypes are better suited for certain cell type-specific functions [14].

Building upon existing research, it has been observed that exposure to ELF-EMFs can enhance the proportion of differentiated neurons and promote the neurite outgrowth of embryonic neural stem cell (eNSC)–derived neurons [15]. Moreover, the expression of the proneural genes, NeuroD and Ngn1, which are crucial for neuronal differentiation and neurite outgrowth, was shown to be increased after ELF-EMF exposure, followed by upregulation of TRPC1 [15]. Another study demonstrated that ELF-EMF exposure enhances spatial learning and memory and increases the number of differentiated neurons [16]. In addition, ELF-EMFs have been shown to promote neuronal differentiation through NMDA receptor activation [17]. This suggests that ELF-EMFs could potentially be used to modulate neuronal function and cognitive behaviors [18].

However, the exact mechanisms by which ELF-EMFs influence β-3 tubulin expression and neuronal differentiation are still not fully understood and more research is needed in this area. It is also important to note that the literature presents a dichotomy in the effects of ELF-EMFs, with some studies highlighting beneficial impacts on neurogenesis and neuronal differentiation, while others point to potential detrimental effects. Our study echoes this dual nature of ELF-EMFs. However, a key insight from our research is the understanding that the nature of these effects is not random but rather contingent on the strength of the applied fields. Furthermore, the duration of application and frequency may also play significant roles. This underscores the nuanced nature of ELF-EMFs’ impact and the importance of considering these factors in future studies.

### 3.2. Pulsating ELF-EMF Exposure and Potential for NSC Differentiation towards the Astrocytic Phenotype

Even though our data suggest that ELF-EMF exposure significantly impacts NSC differentiation towards or away from the neuronal phenotype, our findings also indicate that the effect includes active promotion of neurogenesis. Notably, we observed that ELF-EMFs actively steer NSC differentiation towards various cell lineages, particularly astrocytes. This is evidenced by the upregulation of GFAP expression when exposed to 50Hz pulsating ELF-EMFs of strengths 7.26–13.31 mT (B2) and 13.31–18.15 mT (B3) (*p* ≤ 0.0001), with the effects being more pronounced in group B3 (Figure 2d). In contrast, the application of lower-strength ELF-EMFs (1.21–7.26 mT, B1) resulted in a downregulation of GFAP expression (*p* ≤ 0.05). This implies that the differentiation of NSCs towards astrocytic phenotypes could be a function of ELF-EMF strength, with low-strength ELF-EMFs potentially acting as inhibitors, while high-strength ELF-EMFs may serve as promoters.

Indeed, the differentiation of NSCs into astrocytic phenotypes is a topic that remains underrepresented and largely unexplored in scientific literature. To date, the most notable contribution to this field is a 2014 study by Ma et al., which stands as a solitary beacon in this area [19]. Ma et al.’s research concluded that ELF-EMF exposure did not significantly alter the proportions of neurons (identified by Tuj1-positive cells) and astrocytes (identified by GFAP-positive cells), as determined by immunofluorescence assays [19]. While the study by Ma et al. presents findings that differ from ours, this discrepancy could be attributed to their specific application of ELF-EMFs—a singular strength of 2 mT, 50 Hz for a duration of 3 days. A multitude of studies have indeed demonstrated that the effects observed post-ELF-EMF application are contingent on various parameters such as the frequency, strength, and duration of treatment [18,20]. Given this variability, it is not surprising to encounter contradictory effects. Therefore, further research is imperative to unravel the precise molecular mechanisms that underpin our findings. This will not only reconcile the disparities but also contribute to a more comprehensive understanding of the influence of ELF-EMFs on neural stem cell differentiation.

### 3.3. Pulsating ELF-EMF Exposure and Potential for Induction of NSCs’ Stemness Phenotypes

Interestingly, NES immunoreactivity of NSCs was mostly unaltered post-treatment application, with an exception in group B2 (7.26–13.31 mT), where upregulation in the marker’s expression was visible (*p* ≤ 0.0001) (Figure 2c).

During embryogenesis, NES expression can be found in tissues outside of the central nervous system (CNS), especially in developing muscle. Once these cells become differentiated and cease to divide, NES expression is downregulated, often with the concomitant upregulation of other tissue-specific intermediate filament proteins. This suggests that NES expression could be a key factor in determining the fate of NSCs. Nestin (NES) is a type VI intermediate filament protein that is predominantly expressed in NSCs during development and in the adult brain [21,22]. It plays a crucial role in cellular processes such as stemness, migration, and cell cycle regulation [22]. NES is also considered a biomarker of invasive phenotypes, particularly in glioblastomas [22].

Interestingly, even though the following study was conducted on bone marrow stromal cells, Wislet-Gendebien et al. demonstrated that only NES-positive stromal cells are able to differentiate into GFAP-positive cells when they are co-cultivated with NSCs [23]. They can even migrate to damaged regions [24], which makes NES a promising target for neurological therapies aimed at promoting neuroplasticity, regeneration, and repair of the nervous system.

### 3.4. Molecular Mechanisms Underlying Observed Effects of ELF-EMFs on NSCs

One of the main properties of neural stem cells is their distinct electrophysiological features. During the course of their differentiation, NSCs express specific ion channels depending on the microenvironment. With that in mind, they are susceptible to electromagnetic field influence. As such, some of the main molecular targets for the observed action of EMFs upon neural stem cells include brain-derived neurotrophic factor (BDNF), Ca^2+^ ion channels, transient receptor potential canonical 1 (TRPC1), and the miR-25/p57 signaling pathway.

Studies performed on B6(Cg)-Tyr^c−2J^/J mice after photothrombotic occlusion have shown that LF-PEMF treatment upregulates the BDNF/TrkB/Akt signaling pathway [25], suggesting the treatment’s neuroprotective properties. These findings are supported by recent experiments utilizing a chronic ELF-EMF exposure setup, which induced an increase in BDNF expression in the rat hippocampus [26]. Since the synthesis and release of BDNF is associated with synaptic plasticity and cell survival, upregulation in BDNF expression could enhance neurotrophic processes and increase the resistance to neurodegenerative disorders, promoting healthy aging.

The next molecular target is Ca^2+^. Since one of the main mechanisms of action of electromagnetic fields is that any charged particles in their vicinity experience a Lorentz force, these fields can alter the distribution of ion channels along the cell membrane, control the kinetics of voltage-gated ion channels, and upregulate the expression of voltage-gated calcium channels (VGCCs) [27]. Application of ELF-EMFs to cortical NSCs enhances the functionality of VGCCs, inducing an increase in intracellular Ca^2+^ through CaV1 channels [28]. This causes an increase in CREB phosphorylation, regulating the expression of genes responsible for NSC proliferation and differentiation [28]. This is supported by a growing body of evidence that proposes that the Ca^2+^ signaling pathway controls NSC self-renewal following injury. Additional studies have also demonstrated that ELF-EMFs influence neurons in the Schaffer collateral-CA1 (SC-CA1) region of the rat hippocampus by weakening long-term potentiation and enhancing long-term depression [29]. Interestingly, these beneficial effects of EMFs were attenuated after the blockage of voltage-gated Ca^2+^ channels and calcineurin. This suggests that the effects of ELF-EMFs on synaptic plasticity are, to a great extent, mediated by the Ca2+/calcineurin pathway. Nevertheless, further research is required to understand the precise molecular mechanisms through which EMFs impact the precursor cells within the nervous system.

Equally important to VGCCs are the TRPC1 channels, non-specific cation channels that play a key role in Ca^2+^ and Na+ influx and, consequently, NSC proliferation [30]. Some studies have shown that ELF-EMFs significantly upregulate TRPC1 expression, which, in turn, mediates Ca^2+^ influx and promotes neurogenesis [31]. These findings are supported by recent studies on embryonic neural stem cells which, when exposed to ELF-EMFs, exhibited an upregulation of TRPC1 expression, accompanied by increased peak amplitude of intracellular Ca^2+^ levels [15].

Additionally, ELF-EMFs have also been shown to act upon the miR-25/p57 signaling pathway, which plays a crucial role in enhancing adult NSC proliferation [32]. Through potentially upregulating the expression of miR-25 and inducing a suppression of its target gene p57, ELF-EMFs can promote adult NSC proliferation and inhibit cell cycle arrest.

### 3.5. Limitations of this Study and Future Considerations

One of the drawbacks of this preliminary study is the consistent use of a singular EMF frequency (50 Hz). With that, further experiments are required to define the optimal frequency and strength range that lead to quantifiable changes in protein and mRNA expression, yielding a functional characterization of neural stem cells following EMF application.

Additionally, NSCs are a diverse population, with variations in their differentiation potential, proliferation capacity, and response to external stimuli [33]. This inherent heterogeneity can introduce variability in experimental results. For instance, certain subpopulations of NSCs may preferentially differentiate into astrocytes, while others may be more inclined towards neuronal differentiation [34]. Therefore, the observed results in this preliminary experiment are not solely a reflection of the differentiation potential of NSCs as a whole, but rather the combined output of various distinct subpopulations. Moreover, the microenvironment or “niche” in which NSCs reside can further contribute to their heterogeneity [34,35]. Differences in niche characteristics such as the availability of growth factors, extracellular matrix composition, and cell–cell interactions can influence NSC behavior and differentiation [36]. While the generalized approach undertaken by our study has allowed us to draw certain conclusions about the direction of NSC differentiation, we acknowledge that it does not provide a comprehensive picture of the entire NSC population. To address this limitation, future studies would benefit from strategies that account for this heterogeneity, such as single-cell analysis techniques, to provide a more accurate and comprehensive understanding of NSC biology while under the influence of EMFs.

## 4. Materials and Methods

### 4.1. Animals and Housing

B6(Cg)-Tyr^c−2J^/J, also known as B6 albino mice, were provided by the Experimental Animal Centre of the Croatian Institute for Brain Research at the University of Zagreb School of Medicine. Mice were housed with free access to food and water at 22 ± 2 °C, 55% ± 10% humidity, and a 12/12 h light/dark cycle. All experimental procedures were executed in accordance with approval by the Internal Review Board of the Ethical Committee of the University of Zagreb School of Medicine (approval no. 380-59-10106-17-100/27, 26 January 2017) and the EU Directive 2010/63/EU.

### 4.2. Neural Stem Cells and Cell Culture

Neural stem cells (NSCs) were isolated from the telencephalic wall of B6 albino mouse embryos (E14.5). Isolated cells were placed into T75 flasks containing proliferation-supporting medium comprising DMEM/F-12, GlutaMAX Supplement (Gibco, Waltham, MA, USA), 2% B-27 supplement (Gibco), N-2 supplement (Gibco), 5 mM HEPES, and 1% Penicillin–Streptomycin mixture (Pen-Strep, 5,000 U/mL, Gibco), with added 10 ng/mL recombinant mouse fibroblast growth factor basic (FGF-β, Gibco) and 20 ng/mL epidermal growth factor (EGF, Gibco). These cells were cultivated in suspension, resulting in the formation of neurospheres. Growth factors were added after 2 days and, when neurospheres reached 150–200 μm in diameter, they were dissociated in Accutase (Gibco). Single NSCs were re-plated in T75 flasks at concentration 50,000 cells/mL for passaging.

Following one day of dissociation, passage 3 (P3) cells were seeded in poly-D-lysine (PDL, mol wt 30,000–70,000, Sigma-Aldrich, St. Louis, MO, USA)-coated 12 mm coverslips placed in a 24-well plate. Cells were plated at 30,000 cells per well in 600 µL of DMEM/F-12, GlutaMAX medium containing 5% heat-inactivated FBS (Gibco), and 1% Penicillin–Streptomycin mixture. After 3 days, half of the medium was replaced, and cells were allowed to differentiate for 5 days.

### 4.3. Experimental Design

In this pilot, we applied pulsating ELF-EMFs to B6 albino mouse neural stem cells in vitro (Figure 1a). The electromagnet was designed as a 156-turn (N) solenoid out of an iron-threaded rod and copper wire (16.2 cm length (l), 5.3 cm radius, 0.9 mm wire diameter), generating a horizontal magnetic field. It was powered by an AC/DC converter (230–12 V DC, 15 A, 50 Hz), and a laboratory power supply (input: 6–60V; output: 0–50 V, 0–15 A, 50Hz was used to generate arbitrary triangular waveforms of 10 different amplitudes. The solenoid was placed inside the exposure incubator and the system was programmed in such a way that each amplitude was active for t = 1 s before it moved to the next amplitude. With that, and throughout the experiment, a total of 3 different runs were made, each for different power levels, where every group had 10 levels of different intervals lasting for 1 s. Each run comprised different current ranges (I = 1–6 A, 6–11 A, 11–15 A). The amplitudes were chosen at random within the current ranges for a total of 10 different power intervals per current range. For the purpose of calculating the predicted strength of the electromagnetic field around the solenoid, the following formula $B=\frac{\mu_{0}NI}{l}\;[T]$ was used, where μ_0_ stands for the relative permeability of a vacuum. Thereby, the projected strength of the magnetic field produced by the coil for each run was $B_{1}=1.21–7.26\;mT$; $B_{2}=7.26–13.31\;mT$; $B_{3}=13.31–18.15\;mT$.

### 4.4. Application of Electromagnetic Fields

The EMF apparatus and the well plates containing three technical replicates of cell cultures, after 5 days of differentiation, were placed into an incubator with custom-made shelves and thermoresistant rod holders. In the context of this experiment, which involves the use of direct current (DC), it is important to consider the orientation of the electromagnetic field generated by the solenoid. Specifically, for cultures positioned equidistantly above and below the solenoid, the direction of the electromagnetic field remains consistent. This uniformity in field direction is a direct consequence of the unidirectional nature of the DC used in the experiment. Thus, any effects observed in the cultures can be attributed to this consistent electromagnetic environment. The cells were treated in 1h intervals with a non-thermal EM signal, which consisted of a 1–6 A oscillating carrier pulse. This method was repeated for two additional EMF strengths, corresponding to 6–11 A and 11–15 A oscillating carrier pulses. Cultures assigned to control groups were kept in a separate incubator and exposed to the same conditions in the absence of EM signals.

### 4.5. Immunocytochemistry

Following each run, cells in the control and treatment group were fixed for 10 min at room temperature (RT) with 4% PFA. Afterwards, cells were washed three times with a phosphate buffer, and then 0.2% Triton X-100 in PBS was used to permeabilize cell membranes for 10 min. Blocking of the non-specific binding of antibodies was performed with 3% DS in PBS for 30 min. The cells were then transferred into a humid chamber and incubated with 100 μL of primary antibody solution for (a) glial fibrillary acidic protein (GFAP) (1:1000, Abcam, Cambridge, UK, #ab4674), (b) nestin (NES) (1:200, Milipore, Burlington, MA, USA, #MAB353), and c) β-3 tubulin (1:500, BioLegend, San Diego, CA, USA, #802001) at 4 °C overnight.

After the primary antibody incubation, cells were washed in PBS 3 times for 5 min, incubated with the secondary antibody for 2 h at RT (Alexa Fluor 488, 1:1000; Alexa Fluor 546, 1:1000; and Alexa Fluor 488, 1:1000), washed 3 times, and, subsequently, stained with nuclear stain for 10 min. Coverslips were washed 3 times with PBS and mounted on the Dako mounting medium (Agilent Technologies, Santa Clara, CA, USA). Slides were left to dry overnight at RT in the dark and then stored at 4 °C. A 3% solution of serum in PBS was used to dilute the primary and secondary antibodies, while the cell nuclei were stained with 300 nM nuclear stain 4′,6-diamidino-2-phenylindole (DAPI) in PBS. This procedure was repeated for each of the three runs.

Obtained images were analyzed with a standardized protocol in Cell Profiler (Broad Institute’s Imaging Platform, Cambridge, MA, USA). Before proceeding with image analysis, it was necessary to split the obtained images, which were stored as multi-channel z-stacks. This task was accomplished using Fiji. Firstly, for each multi-channel image, a new folder was created. Then, the image stack was opened in Fiji, and the “Image > Color > Split channels” function was utilized. Subsequently, each resulting channel was saved with a distinct identifier corresponding to the specific staining employed, such as NES, GFAP, β-3 tubulin, and DAPI. Experiments and ICC staining were each performed by a separate group of researchers to enable interpretations of results on the basis of a blinded review. Obtained slides were coded to avoid interpretation bias, yielding randomized primary outcome data. The randomization code was broken after analysis.

### 4.6. Cell Counting

Ten regions of interest (ROIs) were identified for each sample, and automatic cell counting, including colocalization of DAPI with other markers (GFAP, NES, and β-3 tubulin), was performed in Cell Profiler. The total number of cells in each ROI was taken as the total number of DAPI-stained nuclei.

### 4.7. Statistical Analysis

Results were expressed as mean ± SD. A two-way ANOVA with Tukey’s multiple comparisons was performed using GraphPad Prism version 8.00 (GraphPad Software, San Diego, CA, USA). The significance levels were as follows: * *p* ≤ 0.05, ** *p* ≤ 0.01, *** *p* ≤ 0.001, **** *p* ≤ 0.0001. The data collected from the technical replicates were merged into a single dataset for that group. All measurements were normalized to the total number of cells within that ROI where colocalization of DAPI and the specific marker was observed. A graphical depiction of the intensity of each labelling, expressed as mean pixel intensity per cell, was made.

## 5. Conclusions

In conclusion, this pilot study has shed light on the potential of ELF-EMFs to influence the differentiation of NSCs. Our findings indicate that exposure to ELF-EMFs can significantly influence the expression or suppression of neuronal markers in NSCs, exemplified by the immunoreactivity of the neuronal marker β-3 tubulin. Additionally, our observations suggest that ELF-EMFs may also have an impact on the differentiation of NSCs into astrocytic phenotypes, as evidenced by the expression of GFAP (Figure 2d). This suggests that ELF-EMFs could be utilized to initiate neurogenesis and guide NSC differentiation, a finding that aligns with results from previous studies [6,8,28].

The influence of ELF-EMFs on NSCs’ differentiation into astrocytes is a relatively new area of research. Preliminary studies suggest that while ELF-EMFs do not affect the percentages of neurons and astrocytes differentiated from eNSCs [23], they do alter the expression of genes regulating neuronal differentiation. This implies that ELF-EMFs could potentially be used to guide the differentiation of NSCs, including the process of neurogenesis. However, it is important to note that the effects of EMF stimulation are known to be dependent on its intensity, frequency, and duration of exposure. Therefore, additional quantitative studies and gene expression profiling are required to further elucidate the mechanisms behind this approach and to quantify the role of molecular targets, growth factors, and reactive oxygen species in this process.

Continued investigation in this research domain holds promise for advancing EMF-based technologies, potentially enhancing the effectiveness of therapeutic strategies aimed at addressing neurodegenerative and demyelinating disorders, in conjunction with stem cell-based therapies.

## Data Availability

The data that support the findings of this study, including raw and processed data, are available from the corresponding author, J.I., upon reasonable request.
